# Questionnaire results of user experiences with wearable exoskeletons and their preferences for sensory feedback

**DOI:** 10.1186/s12984-018-0445-0

**Published:** 2018-11-23

**Authors:** Heidi Muijzer-Witteveen, Nienke Sibum, Rosanne van Dijsseldonk, Noël Keijsers, Edwin van Asseldonk

**Affiliations:** 1 Biomechanical Engineering, University of Twente, Drienerlolaan 5, Enschede, 7522 NB the Netherlands; 2 Research, Sint Maartenskliniek, Hengstdal 3, Nijmegen, 6574 NA the Netherlands

**Keywords:** Wearable exoskeleton, Spinal cord injury, Sensory feedback, User preferences, Online questionnaire

## Abstract

**Background:**

Wearable exoskeletons can be a powerful tool for the facilitation of ambulation of complete Spinal Cord Injury (SCI) subjects, which has several psychological and physical advantages. However, exoskeleton control is difficult for this group of users and requires a long period of training. People with SCI not only lack the motor control, but also miss the sensory information from below the level of the lesion, which is for example very important in their perception of body posture and makes balancing with an exoskeleton difficult.

It is hypothesized that through sensory substitution part of the missing sensory information can be provided and might thereby improve the control of an exoskeleton. However, it is not known which information would be most important to receive while using an exoskeleton and how this feedback should be provided.

**Methods:**

To investigate the preferences of users of an exoskeleton, a questionnaire was filled out by 10 SCI subjects who underwent a training program with a commercial exoskeleton (ReWalk).

The questionnaire consisted of questions about the use of the exoskeleton to identify which information is missing and which instructions from the therapists were needed to be able to control the exoskeleton. The second part of the questionnaire focused on the possibilities of sensory feedback and preferences for stimulation methods (auditory, vibrotactile or visual) and feedback timing (discrete or continuous) were investigated. Furthermore, six options for feedback parameters (step initiation, continuous and discrete gait phases, foot position and mediolateral and anteroposterior weight shift) were proposed and the respondents were asked to indicate their preferences.

**Results:**

Three feedback parameters (feedback about mediolateral and anteroposterior weight shift and feedback about step initiation) were considered as possibly helpful by the respondents. Furthermore, there were slight preferences for the use of vibrotactile (over auditory and visual) and discrete (over continuous) feedback.

**Conclusions:**

The answers of the respondents on the optimal feedback parameters were rather variable and therefore it is recommended to let the users choose their preferred feedback system during a training session with several feedback options. However, there are slight preferences for the use of vibrotactile stimulation provided in a discrete way.

**Electronic supplementary material:**

The online version of this article (10.1186/s12984-018-0445-0) contains supplementary material, which is available to authorized users.

## Background

Over the last few years, wearable exoskeletons used to facilitate ambulation of people with spinal cord injury (SCI), have developed quickly [1–4] and resulted in the introduction of some exoskeletons to the market [5–9]. Currently these exoskeletons are mainly used in rehabilitation centers. Several of these centers have developed programs to train people with SCI how to use the exoskeletons.

An intensive and time consuming training program is required before exoskeleton use without assistance by others is possible for people with complete motor SCI [10, 11]. The complex learning process is due to the disrupted nervous tract in the spinal cord, which impacts the control of the users’ legs, but also the sensory information back to the user. Current exoskeletons take over the disrupted control of the legs and some of them even provide information about the status of the exoskeleton back to the user [5, 6], but they do not provide the complex sensory input of natural proprioception and tactile sensation. In combination with the visual and vestibular inputs, these somatosensory inputs play a very important role in human balance [12]. Although sensory reweighting takes place after the loss of the somatosensory input [13], one can expect that the lack of somatosensory input will negatively influence the capabilities of these patients in balancing with and controlling an exoskeleton.

In a preprogrammed gait, the level of weight shift is important to prepare for a step and create enough foot clearance. However, accurate weight shift control is difficult when the perception of the weight distribution is disturbed due to the lack of somatosensory input from below the level of the lesion. This is likely one of the reasons why learning to walk with an exoskeleton is time consuming and difficult [10, 11] for people with SCI. It is hypothesized that by providing some sensory information, the control of the exoskeleton can be improved. Furthermore, (sensory) feedback plays an important role in the learning of motor tasks [14] and adding sensory feedback will therefore improve the learning process of the exoskeleton control.

In the field of prosthetics, in which sensory information is also missing, questionnaires have been used to derive user preferences for future upper-limb prostheses [15–17]. The addition of sensory feedback was considered as one of the major future improvements in prosthetics. In studies with lower-limb amputee subjects and sensory feedback, the applied stimulation methods vary largely [18–24]. Besides the choice for a stimulation method, also different feedback parameters were investigated in these studies [18–24]. The sensory feedback usually resulted in a better gait symmetry [19, 21–23] or increased confidence during walking [18, 20]. The described stimulation methods can also be applied in wearable exoskeletons, but the optimal feedback parameters will most likely differ, because the focus for prosthetics is on the prosthetic leg use and restoring symmetry, while for wearable exoskeletons both legs are involved.

Only a few studies have investigated the application of sensory feedback in wearable exoskeletons [25–28]. And only in the study of De Castro [27], a limited number of SCI subjects were involved, who could use the feedback and walk without continuously looking at their legs. Similar to the lower-limb prostheses, the stimulation methods in the various studies varied (pressure cuff [25], electrostimulation [26, 27] or haptic force feedback [28]) as well as the feedback parameters (knee torque [25], knee angle [25], hip angle [26], swing phase [27] and force under the feet [28]). There is a clear need for sensory feedback in wearable exoskeletons, but it is not clear which stimulation methods and which feedback parameters should be used. To our knowledge, there are only a few studies that involved questionnaires to evaluate stakeholder perspectives of exoskeleton technologies [29–31], but sensory feedback was not the main focus of these studies. In the study of Gagnon et al. the learnability of exoskeleton use was evaluated, amongst others, by asking whether the sound of the exoskeleton and the instructions by the therapists were helpful. Both questions were answered positively: 77 and 97% respectively on a scale of 0 to 100 [30].

The goal of this study was to evaluate current experiences with wearable exoskeletons and the potential of sensory feedback from the user point of view. The experienced and most prominent shortcomings during exoskeleton walking were identified, the potential of multiple stimulation methods was estimated and the expected usefulness of several feedback parameters was assessed through an online questionnaire filled out by 10 SCI users of an exoskeleton.

## Methods

### Subjects

A link to the online questionnaire was distributed via email to 14 subjects who had been participating in a study with the ReWalk wearable exoskeleton at the Sint Maartenskliniek in Nijmegen, the Netherlands, in the period from September 2015 to September 2017. All participants gave written informed consent in accordance with the Declaration of Helsinki. The study was approved by the medical ethics committee of Arnhem-Nijmegen (2016–2418) and the internal review committee of the Sint Maartenskliniek. 10 out of the 14 subjects completed the questionnaire (see Table 1 for the characteristics). They had been using the exoskeleton for at least 2 training sessions (of more than 1 h).

**Table 1:** Characteristics of the 10 SCI respondents. All were involved in the training program with the ReWalk exoskeleton

Male /female	Age (years)	Neurological lesion level	ASIA-score	Time since injury onset (years)	Number of 1.5-h training sessions	Period of home use (weeks)	Self-reported experience with exoskeleton (0–5)
Female	46	T5/6	A	24	28	2	5
Male	26	T9	A	5.5	23	2	4
Male	29	T4	A	10	24	0	4
Female	26	T11	A	6	22	0	5
Female	46	T12	A	12	28	9	5
Male	48	T4/T5	A	17	2	0	1
Female	49	T4	A	5	28	0	3
Male	41	T12	A	7	24	2	4
Male	30	T10	A	4.5	19	2	3
Female	42	T9	A	17	12	0	3

The level of experience with the exoskeleton varied over the subjects. One subject had to quit the training after two sessions due to physical problems and another subject had not yet finished the complete training program. Besides the number of hours of training with the exoskeleton and the duration of home use, the respondents also provided a score on a scale of 0 (hardly any experience) to 5 (a lot of experience) for the self-reported experience with the exoskeleton.

### Questionnaire

An online questionnaire was composed via Google forms, consisting of 16 questions regarding the use of sensory feedback during walking in an exoskeleton (see Additional file 1). The questions were formulated by the investigators, in close collaboration with two therapists who checked whether the questions were clear, useful and complete. The first five questions were related to the *current exoskeleton use* as experienced during the training and involved the problems that occurred during the training, the instructions that were given by the therapists, the instructions that helped the most, the tricks they learned during training and what information the subjects missed during the training. The next two questions were related to the *stimulation methods* that can be used to provide the feedback: 1) which stimulation method they would prefer (vibrotactile, auditory or visual) and 2) whether they were using the sound that the exoskeleton made during the training session (yes or no). Subsequently, 6 different *feedback parameters* were suggested: 1) feedback about step initiation, 2) continuous feedback about gait phases, 3) discrete feedback about gait phases, 4) feedback about foot position, 5) feedback about mediolateral weight shift and 6) feedback about anteroposterior weight shift. For each feedback parameter an explanatory figure was included for clarification. Subjects indicated for each parameter whether feedback about this parameter would be helpful on a 5-point scale (“not at all”, “not much”, “neutral”, “a little” or “a lot”) for them during walking in the exoskeleton. Finally, subjects’ preferences for the *timing of feedback* were investigated. In relation to the feedback about different gait phases, they were asked to indicate at which moment of the gait cycle (heel strike, foot flat, midstance, heel off, toe off, mid swing) they would like to receive feedback and more in general whether they would prefer continuous or discrete feedback. For all questions the answers could be left open (open questions) or the option “no idea” could be checked (multiple-choice questions) and for each multiple-choice question there was space for the subjects to provide additional information. Subjects were given the chance to bring in other issues that were not discussed in the last question of the questionnaire.

Open questions were used in the first part of the questionnaire to give the exoskeleton users the freedom to write down all the possible issues that they might have encountered, while for the remaining parts of the questionnaire closed questions were used to be able to compare the answers between the respondents.

The subject responses were collected through the Google forms application and downloaded for analysis. The answers to the open questions and the additional comments given for the multiple choice questions were put together per question and overlapping answers were identified. The different options for the gait feedback parameters were taken together and summarized in one stacked bar plot to compare between the different feedback options. A feedback option was seen as useful when the majority of the respondents rated it as “a little” or “a lot” helpful.

## Results

### Current exoskeleton use

The responses of the subjects to the general, open questions (1–5) about the use of the wearable exoskeleton during training showed hardly any overlapping answers over the respondents. The following problems that occurred during the training were mentioned (unique answers): technical problems with the remote controlling wristband, system errors of the exoskeleton, the lack of being able to adjust step length, the occurrence of skin damage, difficulties with determining the body position, difficulties in walking over uneven grounds, difficulties in walking on small slopes and for one of the subjects no problems occurred during the training.

The most common instructions given by the therapists, mentioned by four respondents, were: 1) keep the back straight, 2) do not throw the trunk forwards or buttocks backwards, 3) move the hips forwards and 4) perform a good weight shift from left to right. Instructions related to crutch placement were mentioned by three respondents: 1) place crutches at the right position, 2) don’t lean too much on the crutches and 3) put the crutches on the ground as silent as possible. A clear roll-over of the feet was mentioned twice. Other instructions were: feel what the exoskeleton is doing and let the exoskeleton move you. Only one or two instructions were mentioned per respondent, and therefore there were no specific instructions that were considered the most helpful (next question), the respondents just copied their answers from the previous question or rephrased them.

The exoskeleton users were also asked to indicate which tricks they used to improve their control of the exoskeleton. Three respondents did not use any tricks and for the others the answers to this question were overlapping with the responses to the questions about the instructions given by the therapists: get rhythm during crutch placement, swing the hips forward, contract the belly muscles to make pelvis tilt and walking easier, pull yourself forward with the arms in the crutches to keep your body upright, don’t be scared and focus on pelvis shift to the left (right went easy). One of the respondents commented that no specific tricks could be remembered, but “when I realized that I had to cooperate with the exoskeleton it went much better”.

Five respondents indicated that they missed some information during the training with the exoskeleton. For three of them, information on the moment of foot contact (mentioned twice) and body position (gait phase, mediolateral weight shift and overall body posture) would have helped them during walking in the exoskeleton. For another respondent, information of the reason why an involuntary stop of the exoskeleton occurred would have been helpful. And the last respondent mentioned that he would have liked an exoskeleton that could do more by itself during walking and during standing up.

### Stimulation methods

Nine out of ten respondents indicated that during training with the exoskeleton, they did benefit from the sounds that the exoskeleton made during walking.

**Fig. 1:**
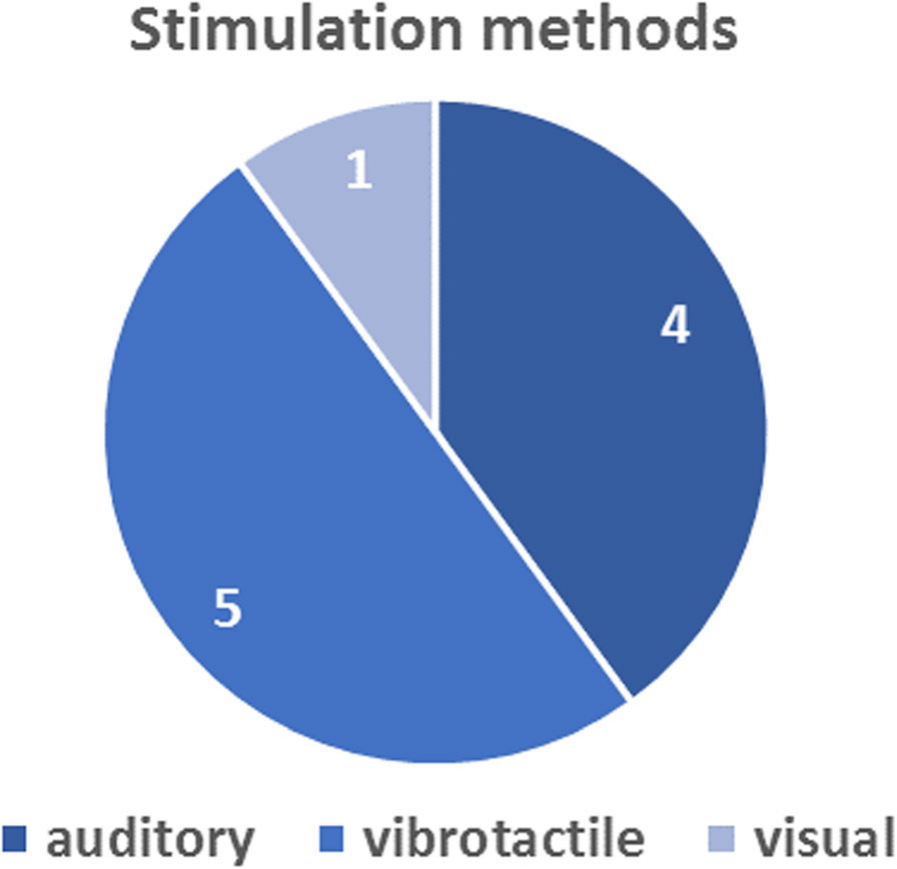
User preferences of the 10 respondents for stimulation methods. Respondents were forced to choose between the three options (auditory, vibrotactile or visual)

Five of the 10 respondents would prefer to receive vibrotactile feedback, 4 respondents preferred auditory and 1 visual feedback (see Fig. 1). Although respondents could only select one single option, two respondents, who preferred the auditory feedback, additionally mentioned that they would prefer a combination of auditory and vibrotactile feedback.

Two respondents also indicated that auditory information may not be useful in each condition. Especially in a noisy environment, which was also the experience of one of the respondents: “The sound of the exoskeleton made me walk in a ‘flow’. I’ve been walking outside once there were two people working with a leaf-blower, which made walking absolutely more difficult”.

### Feedback parameters

**Fig. 2:**
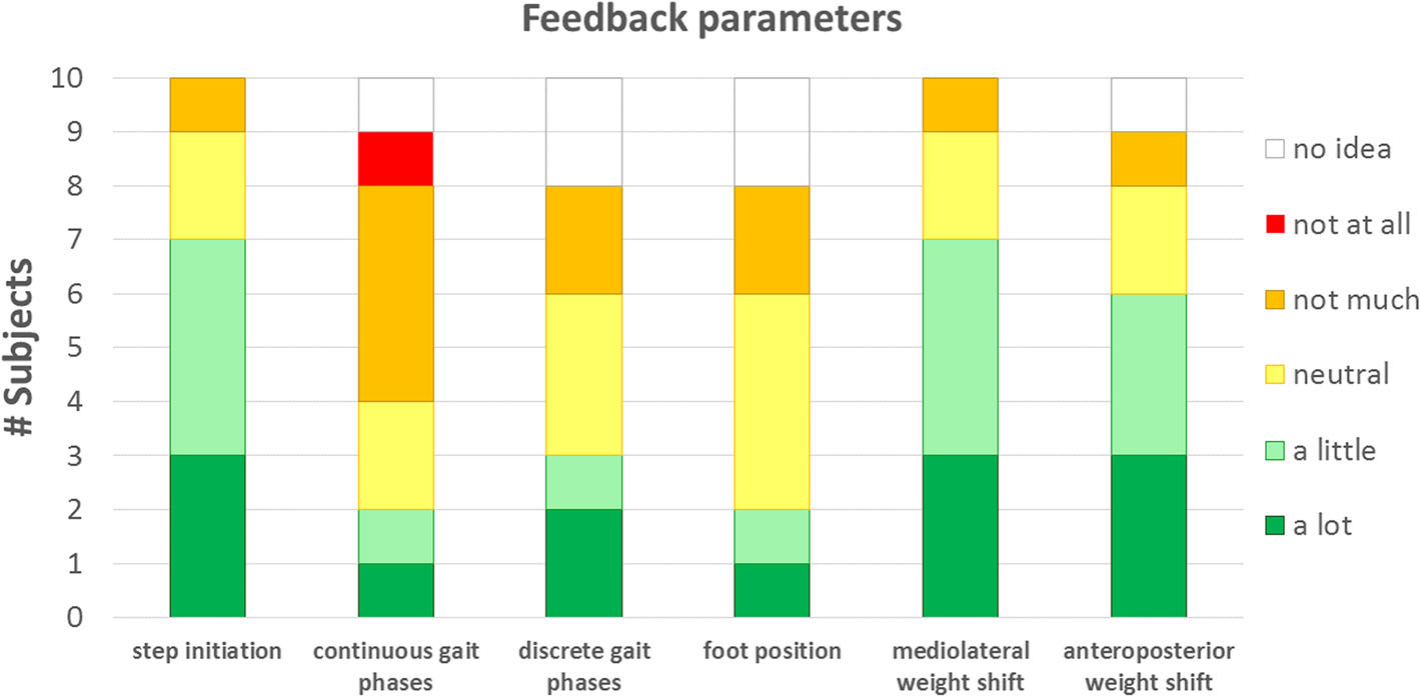
Indicated usefulness of the 6 possible options for gait feedback parameters on a 5-point scale, ranging from “not at all” to “a lot”

The responses indicating the expected usefulness of feedback about 1) step initiation, 2) continuous feedback about the gait phase, 3) discrete feedback about the gait phase, 4) feedback about the foot position, 5) feedback about mediolateral weight shift and 6) feedback about anteroposterior weight shift have been summarized in Fig. 2.

Three of the feedback parameters were considered helpful by more than half of the respondents: feedback about 1) the step initiation, feedback about the 2) mediolateral and 3) anteroposterior weight shift (6 or more votes for “a little” or “a lot”). The positive responses for feedback on step initiation were further emphasized by the comments of the respondents: “You will know that the exoskeleton is in the right mode and a step will take place”, “I think I will like it when I get feedback when the exoskeleton will make a step, especially in unexpected situations” and “Because you can then lean on your hindmost leg”. Feedback about the weight shift was also indicated as helpful by most of the respondents, in which the mediolateral weight shift received 1 vote more than the anteroposterior weight shift (7 to 6 votes respectively). One of the respondents who indicated that feedback about anteroposterior weight shift will not be helpful commented that you would be able to feel the moment of falling forwards or backwards yourself and therefore do not require additional feedback. For the feedback about the mediolateral weight shift, one of the respondents commented that it might also be useful to provide feedback when the weight shift is too large, because she was afraid to fall when moving too much, while usually the movements were not large enough to create enough space for a step. The option to provide feedback when you exceed a safety boundary was also mentioned by another respondent at the last open question. The fact that you don’t know exactly what your body position is, was mentioned twice as a reason to prefer weight shift feedback of both types (mediolateral and anteroposterior): “It is really peculiar that you feel that something goes wrong, but you don’t know why”.

Foot position feedback and feedback about the different gait phases (in continuous or discrete way) were not considered useful by the majority of the respondents. Only 2 of the 10 respondents indicated that foot position feedback could be helpful and in the comments there were no (positive) aspects mentioned regarding this feedback possibility. Only two and three positive votes (for continuous and discrete respectively) were given for feedback about the different gait phases. In accordance to the responses about the step initiation feedback, three respondents mentioned that only a short feedback signal at the beginning (2) or at the end of a step (1) was preferred over feedback about each specific gait phase. As mentioned by two of the respondents, a feedback signal for each gait phase might even easily become annoying. According to one of the respondents the gait phase feedback could be helpful to get used to the rhythm of the exoskeleton, which would however not be needed anymore after a certain amount of training time.

### Feedback timing

**Fig. 3:**
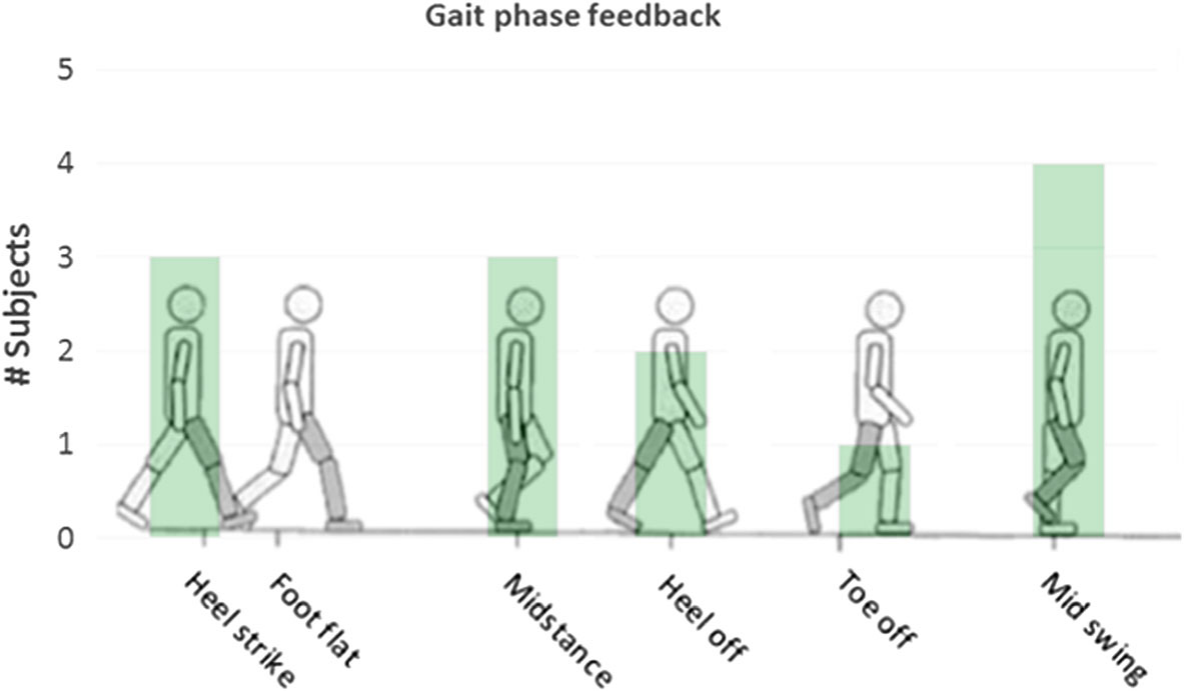
Number of subjects, per specified phase in the gait cycle, who indicated that feedback about the occurrence of that gait phase would be useful. Respondents could select more than one option

Except for the foot flat on the ground, all other 5 possible gait phases were ticked by the respondents as gait phases they prefer to receive feedback about (see Fig. 3). One of the respondents commented that the gait phases that “happen behind your back” are the most important, because you cannot easily see them. A respondent who selected midstance as the most important phase commented that in this phase you have to prepare for the next step and place your crutches forward. Two of the respondents who selected heel strike as the most important gait phase commented that this moment is most important, because you have to prepare for a next step and move your crutches forward or because you have to move your weight to the leading leg.

Out of ten respondents, seven preferred discrete feedback over continuous feedback and the three others preferred continuous feedback. The respondents who preferred discrete feedback commented that with discrete feedback the focus can be more on other things instead of the feedback and that continuous feedback can become annoying after a while. On the other hand the respondents who preferred the continuous feedback commented that you can always feel the feedback and won’t miss important changes when you are distracted for a while.

## Discussion

It is expected that SCI users of a wearable exoskeleton would benefit from additional, artificial sensory feedback, but it is not clear how and which sensory information should be provided back. This paper is a first attempt to provide some insights, from a user perspective, in the shortcomings of current wearable exoskeletons and the possibilities of providing sensory feedback during walking in a wearable exoskeleton. As this study is a preliminary study with a small group of exoskeleton users who only trained with the ReWalk exoskeleton, the conclusions from this study should be treated with care, but still provide useful directions for future work on exoskeletons.

We only approached SCI subjects who had already trained with a wearable exoskeleton. Using this experience, they can better indicate what information is missing during walking and training with current wearable exoskeletons and what sensory information would be helpful.

For all questions regarding the experiences with the exoskeleton and current exoskeleton use, the answers were diverse and a high variability was seen between respondents. Furthermore, there was also no clear preference for one of the proposed feedback parameters or in the selection of the preferred moment of feedback during gait. We don’t expect large differences in the outcomes when increasing the number of respondents, but a significantly larger subject group might lead to more detailed insights, analyses of subgroups and trends that are not visible yet. Based on the diversity of the responses that was already seen, it is suggested to tailor the sensory feedback to the needs and wishes of the individual users of a wearable exoskeleton and evaluate the effect based on each individual’s needs.

Although the responses were diverse on most questions, almost every respondent (9/10) was using the sounds of the exoskeleton, which is in addition to vision the only available feedback source in current exoskeletons. This finding aligns to the results of Gagnon et al. [30], where SCI users also agreed that sounds of the exoskeleton helped them during walking. As the respondents are accustomed to use the sounds of the exoskeleton, this might explain why some of them selected auditory cues as the preferred feedback option over visual or vibrotactile stimulation. However, most respondents (5) would prefer vibrotactile stimulation over auditory feedback, underlining the potential of vibrotactile feedback. Some respondents might have voted for vibrotactile feedback due to the fact that some examples with vibrotactile feedback were given in the questionnaire. However, all of them were already familiar with some kind of auditory feedback. Furthermore, some respondents with a preference for auditory feedback also indicated that this auditory feedback would probably not be useful in noisy environments. This was already experienced by at least two of the users of the exoskeleton. The use of vibrotactile feedback is therefore recommended over auditory feedback to circumvent problems with interfering environments and to be able to use the feedback during all daily life activities, like walking outside.

As the proposed feedback options were only described on paper and could not be directly experienced by the exoskeleton users, it might have been difficult to get a clear picture of how the feedback could help them. As a consequence, some respondents voted “neutral” (± 2/10 per question) or “no idea” (± 1/10 per question) for the helpfulness of the feedback parameters. However, none of them always answered with ‘no idea’ or ‘neutral’ for all questions, which indicates that all respondents could imagine for at least one feedback option how it could help them during walking in an exoskeleton. Furthermore, all respondents provided additional comments for at least one of the feedback parameters to underline their preferred feedback option, which shows that they could indeed distinguish between the different options.

Three feedback options received a clear positive response (“a little” or “a lot” helpful) by the majority of the respondents: feedback about step initiation and feedback about the mediolateral and anteroposterior weight shift. These types of feedback are related to the control mechanism of the ReWalk exoskeleton where each step starts automatically in a fixed timing pattern. Therefore, it is important to be completely prepared before the next step starts, which includes crutch placement and weight shift. Feedback at the moment of step initiation would therefore be helpful for the respondents to ensure that they are ready for stepping. This is already incorporated in the ReWalk exoskeleton through a short beep. Furthermore, a certain amount of weight shift is needed to be able to lift and swing the foot and ensure balance. The exoskeleton stops immediately when the amount of weight shift is insufficient. Some of the respondents indicated that they often encountered this problem, because they usually overestimated the amount of their weight shift and would therefore certainly benefit from the feedback.

Despite the limiting fact of this study that the results are solely based on the user experiences with the ReWalk, it is expected that the conclusions drawn from the questionnaire results can also be applied to other exoskeletons. This holds especially for those exoskeletons where the step initiation is triggered by a certain amount of weight shift of the user [5, 6]. Step initiation through weight shift is commonly implemented in exoskeletons [2], because it is rather intuitive and partly resembles the natural way of step initiation.

To our knowledge this is the first time that preferences of exoskeleton users have been investigated in relation to the possibilities of the application of sensory feedback. Stakeholder perspectives of exoskeleton users have been investigated before [29–31], but sensory feedback was not a major aspect in these studies, which was also the case for the related field of lower-limb prostheses [32]. Not taking into account the user preferences resulted in a lot of different feedback methods that are being evaluated in studies on lower-limb prostheses [18–23] and did not lead to any consensus on for example the stimulation method or the use of continuous [18–20, 22] versus discrete [21, 23] feedback.

## Conclusions

Based on the preferences of ReWalk exoskeleton users in this study, weight shift or step initiation feedback through vibrotactile stimulation in a discrete way is proposed for implementation in future, analogous, wearable exoskeletons. As there is also considerable variability in preferences between the exoskeleton users, we recommend offering different feedback options during a few training sessions and letting the user choose a feedback preference.

## Additional file

Additional file 1: Questionnaire. (ZIP 858 kb)

## Abbreviations

ASIA scale: American Spinal Injury Association scale
SCI: Spinal Cord Injury
