# Supplementary material for: Questionnaire results of user experiences with wearable exoskeletons and their preferences for sensory feedback
Source: J Neuroeng Rehabil. 2018 Nov 23;15:112. doi: 10.1186/s12984-018-0445-0 (PMC6260663; doi:10.1186/s12984-018-0445-0)
Supplement: Supplementary file 1 — Questionnaire. (ZIP 858 kb) [file 12984_2018_445_MOESM1_ESM.zip › renamed_64d32.pdf]

Vragenlijst patiënten Nederlands

Lees de introductie voor het beantwoorden van de vragen, zodat u de vragenlijst zo goed mogelijk kan invullen. Vul eerst uw gegevens in.

1. **Geslacht:**
   Markeer slechts één ovaal.
   - Man
   - Vrouw

2. **Leeftijd:**

3. **Hoogte van uw laesie:**

4. **Mate van ervaring met het exoskelet:**
   Markeer slechts één ovaal.

	1	2	3	4	5	
Bijna geen ervaring						Veel ervaring

Introductie vragenlijst

Tijdens de vragenlijst worden er 16 opzichzelfstaande vragen gesteld. Lees alstublieft eerst de hele vraag voor u hem beantwoordt. Soms worden vragen opgevolgd door een korte uitleg zodat u de vraag beter begrijpt. We vragen u om de vragen zo goed en volledig mogelijk in te vullen. Sommige zijn open vragen en sommige zijn meerkeuzevragen. Na veel meerkeuzevragen, wordt u gevraagd om uw antwoord uit te leggen, zodat wij uw keuze beter kunnen begrijpen. Kleur één vakje in bij elke meerkeuzevraag om de vraag te beantwoorden, tenzij anders vermeld.

Het grootste deel van de vragen gaat over feedback die toegepast wordt op personen tijdens het lopen in een exoskelet. Met ‘feedback’ bedoelen we informatie, over bijvoorbeeld het moment van voetcontact, die verstrekt wordt via vibraties of auditieve (bijv geluiden) of visuele informatie. De voorbeelden in de vragen zijn aan de hand van vibraties. Verschillende voorbeelden waarover feedback gegeven kan worden, worden besproken in de vragen.
In het eerste deel van de vragenlijst gaan de vragen over het gebruik van en omgang met het exoskelet. Daarna gaat het over informatie en feedback over loopfasen. Als laatst wordt ingegaan op andere informatie die door feedback verstrekt kan worden.

Algemeen gebruik van het exoskelet

5. **Vraag 1:**
Welke problemen ervaarde u de laatste keren dat u in een exoskelet liep? Probeer kort te antwoorden alstublieft.

6. **Vraag 2:**
Welke instructies krijgt u van de therapeut tijdens de training om uw balans en lopen te verbeteren? Ga hierbij uit van de periode na de eerste paar trainingen. Probeer kort te antwoorden alstublieft.

7. **Vraag 3:**
Welke instructie helpt het meest? Licht uw antwoord toe.

8. **Vraag 4:**
Welke trucjes heeft u zichzelf aangeleerd om beter te lopen in het exoskelet? Licht uw antwoord toe.

9. **Vraag 5:**
Welke informatie over het exoskelet of uzelf zou u tijdens het lopen in het exoskelet graag hebben ontvangen om het lopen in het exoskelet te verbeteren? (U kunt denken aan feedback over hoeveel u naar één kant leunt, het begin/einde van een stap etcetera.)

10. **Vraag 6:**
Welk soort feedback denkt u dat het meest behulpzaam is, vibrotactiele, auditieve of visuele? Vibrotactiele feedback: bijv. vibraties op de borstkas. Auditieve feedback: bijv. piep geluidjes. Visuele feedback: bijv. ledlichtjes.
Markeer slechts één ovaal.

- Vibrotactiele
- Auditieve
- Visuele
- Anders: ____________________

11. Licht uw antwoord toe:

____
____
____
____
____

12. **Vraag 7:**
Helpen de geluiden die gemaakt worden door het exoskelet u tijdens het oefenen? Kunt u bijvoorbeeld horen dat het been bewogen wordt waardoor u weet wat de stand van uw been is?
Markeer slechts één ovaal.

- Ja
- Nee

13. **Vraag 8:**
Stel u krijgt feedback door middel van trillingen. Hoeveel helpt het als er een trilling geven zou worden op het moment dat het exoskelet een stap gaat maken?
Markeer slechts één ovaal.

- Helemaal niets
- Niet veel
- Neutraal
- Een beetje
- Veel
- Geen idee

14. Licht uw antwoord toe:

____
____
____
____
____

Feedback over loopfasen

In Figuur 1 is een loopcyclus te zien, die bestaat uit verschillende loopfasen. Er kan feedback gegeven worden die de huidige loopfase aangeeft (met bijvoorbeeld veranderingen in de intensiteit van de feedback) gedurende het lopen in het exoskelet.

Figuur 1:

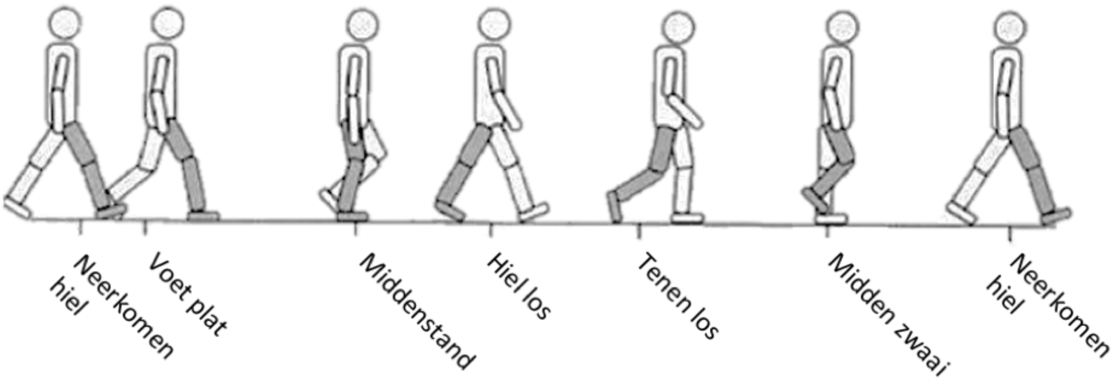

15. **Vraag 9:**

Stel u krijgt feedback over uw loopfasen door middel van trillingen. Bij verschillende loopfasen horen verschillende intensiteiten van trillen. Hoeveel helpt het als het exoskelet continue feedback zou geven over loopfasen? Continue feedback bestaat uit een doorlopend signaal die de huidige loopfase aangeeft, zie Figuur 2. U voelt dus continu een trilling die varieert in intensiteit (harder/zachter) met de loopfase waarin u bent.
Markeer slechts één ovaal.

- Helemaal niets
- Niet veel
- Neutraal
- Een beetje
- Veel
- Geen idee

Figuur 2:

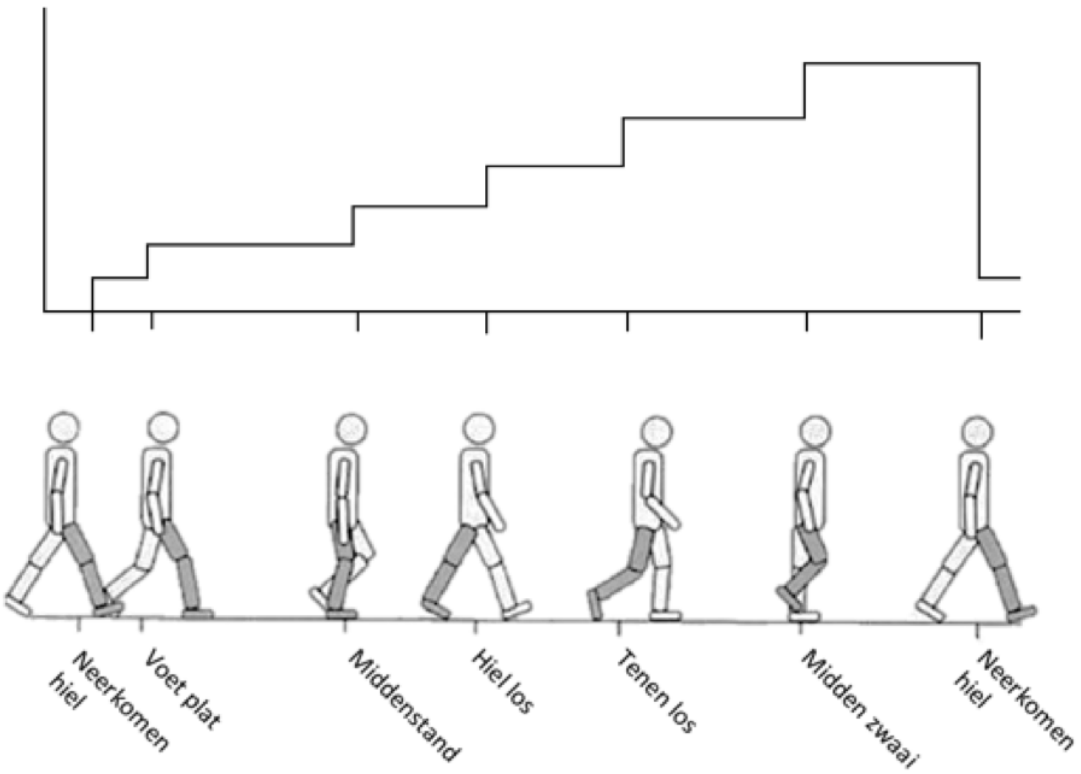

16. Licht uw antwoord toe:

17. **Vraag 10:**
Stel u krijgt feedback over uw loopfasen door middel van trillingen. Bij verschillende loopfasen horen verschillende intensiteiten van trillen. Hoeveel helpt het als het exoskelet discrete feedback zou geven over loopfasen? Discrete feedback bestaat uit losse kortdurende signalen die het begin van de nieuwe loopfase aangeven, zie Figuur 3.
Markeer slechts één ovaal.

- Helemaal niets
- Niet veel
- Neutraal
- Een beetje
- Veel
- Geen idee

Figuur 3:

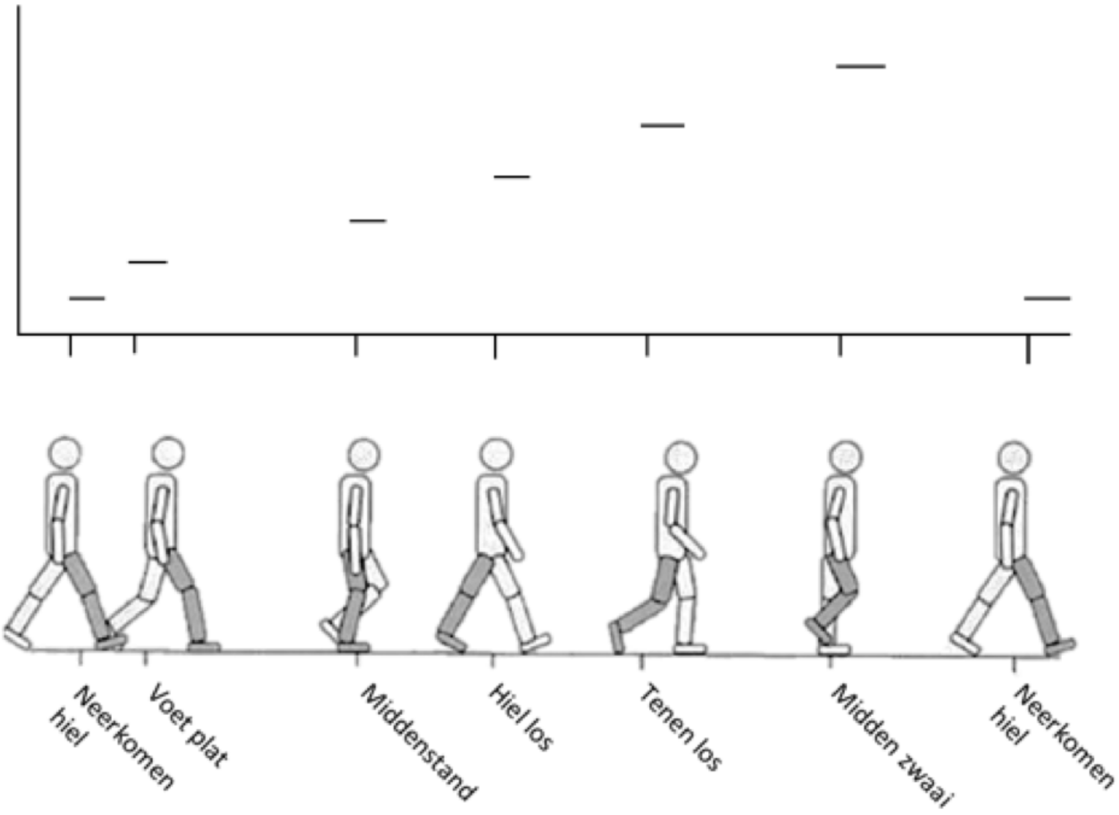

18. Licht uw antwoord toe:

19. **Vraag 11:**
Welk soort feedback denkt u dat het meest behulpzaam is, continue of discrete feedback (zie Figuur 2 en Figuur 3)?
Markeer slechts één ovaal.

- Continue
- Discrete

20. Licht uw antwoord toe:

21. **Vraag 12:**
Over welke loopfasen denkt u dat het belangrijk is om feedback te krijgen (zie Figuur 4)? U kunt meerdere antwoorden geven.
Vink alle toepasselijke opties aan.

- [ ] Neerkomen hiel
- [ ] Voet plat
- [ ] Middenstand
- [ ] Hiel los
- [ ] Tenen los
- [ ] Midden zwaai

Figuur 4:

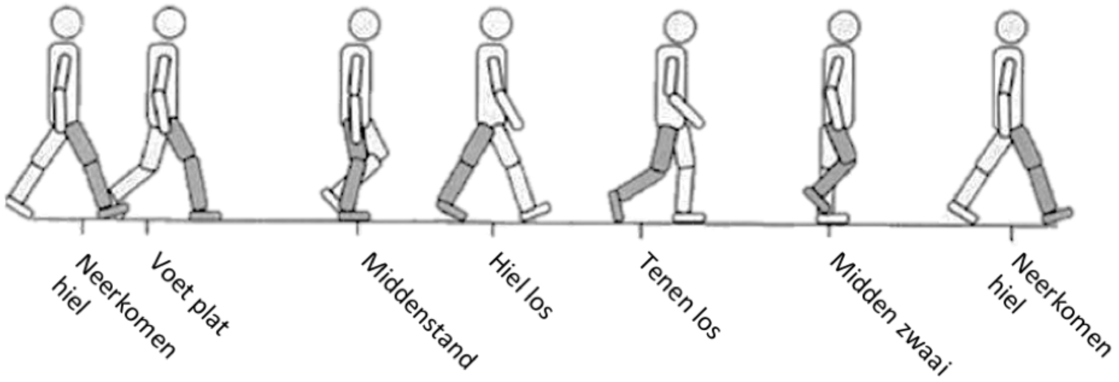

22. Licht uw antwoord toe:

____

____

____

____

____

Andere informatie verstrekt door feedback

23. **Vraag 13:**
Stel u krijgt feedback tijdens het lopen in het exoskelet door middel van trillingen. Deze trillingen geven informatie over de positie van uw voet tijdens een stap. Hoe verder uw voet zwaait, hoe hoger de intensiteit van het trillen. Hoeveel helpt het als het exoskelet feedback zou geven over het zwaaien van uw voet, vanaf het loslaten van de tenen tot het neerkomen van de hiel (zie Figuur 5)?
Markeer slechts één ovaal.

- ( ) Helemaal niets
- ( ) Niet veel
- ( ) Neutraal
- ( ) Een beetje
- ( ) Veel
- ( ) Geen idee

Figuur 5:

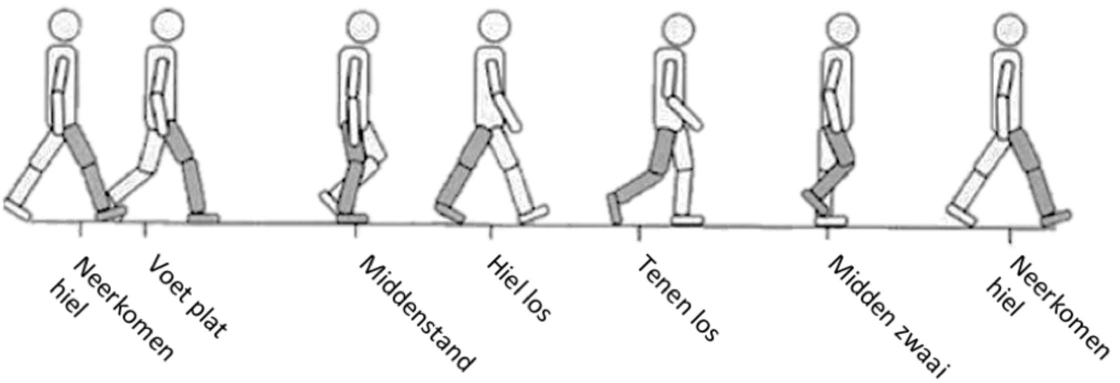

24. Licht uw antwoord toe:

25. **Vraag 14:**
Stel u krijgt feedback tijdens het lopen in het exoskelet door middel van trillingen. Deze trillingen geven informatie over uw gewichtsverplaatsing van de ene naar de andere zijde (bijv. van links naar rechts, zie Figuur 6). Wanneer uw gewicht is verplaatst naar 1 zijde, kan het been aan de andere zijde naar voren zwaaien. Hoe groter de verplaatsing, hoe hoger de intensiteit van het trillen. Hoeveel zou dit u helpen in het omgaan met het exoskelet?
Markeer slechts één ovaal.

- Helemaal niets
- Niet veel
- Neutraal
- Een beetje
- Veel
- Geen idee

Figuur 6:

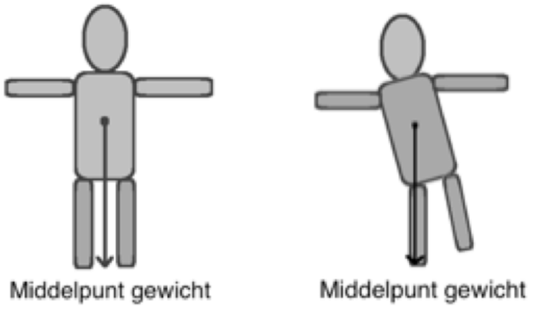

26. Licht uw antwoord toe:

27. **Vraag 15:**
Stel u krijgt feedback tijdens het lopen in het exoskelet door middel van trillingen. Deze trillingen geven informatie over uw gewichtsverplaatsing van voor naar achteren (zie Figuur 7). Hoe groter de verplaatsing, hoe hoger de intensiteit van het trillen. Hoeveel zou dit u helpen in het omgaan met het exoskelet?
Markeer slechts één ovaal.

- Helemaal niets
- Niet veel
- Neutraal
- Een beetje
- Veel
- Geen idee

Figuur 7:

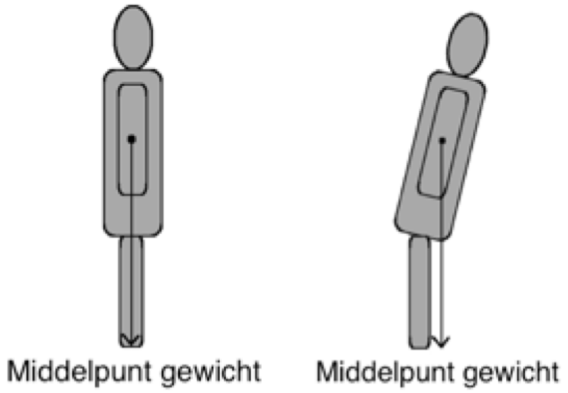

28. Licht uw antwoord toe:

29. **Vraag 16:**

Is er nog iets wat u wilt toevoegen aan deze vragenlijst? Of zijn er tijdens het invullen van deze vragenlijst misschien ideeën bij u opgekomen, over bijvoorbeeld informatie verstrekt door feedback?

Mogelijk gemaakt door Google Forms
