# Supplementary material for: Questionnaire results of user experiences with wearable exoskeletons and their preferences for sensory feedback
Source: J Neuroeng Rehabil. 2018 Nov 23;15:112. doi: 10.1186/s12984-018-0445-0 (PMC6260663; doi:10.1186/s12984-018-0445-0)
Supplement: Supplementary file 1 — Questionnaire. (ZIP 858 kb) [file 12984_2018_445_MOESM1_ESM.zip › Questionnaire English (translated).pdf]

Appendix 1: Questionnaire SCI patients

Read the introduction before answering the questions in order to provided us with your best answers. First, enter your data.

Sex: O Male O Female
Age:
Location of you lesion:
Amount of experience with the exoskeleton:
No experience O O O O O A lot of experience

Introduction questionnaire

During this questionnaire, 16 questions will be asked, which are all standalone. Please read the whole question before answering. Questions are sometimes followed by a brief explanation to help you understand the question better. We ask you to answer the questions as accurate as possible. Some are open questions and some are multiple-choice questions. After each multiple-choice question, you will be asked to explain yourself so we can understand your choice better. For the multiple-choice questions, please check only one box by colouring it in.

The major part of questions is about feedback which will be applied to subjects while walking in an exoskeleton. With 'feedback' we mean information about, e.g. moment of foot contact, provided through either vibrations or auditory (e.g. beeps) or visual cues. The example in the questions are on the basis of vibrations. Different examples for which feedback information can be given will be discussed in the questions.
The first section of the questionnaire is about the use and handling of the exoskeleton. Next, the questions will be about information and feedback regarding gait phases. Finally, other information provided by feedback will be discussed.

General use of the exoskeleton

Question 1: What problems do you experience during walking in an exoskeleton? Please, try to be brief.

Question 2: During practice, what instructions are given to you by the therapists to help you improve your balance and walking when necessary? Use the period after the first trainings. Please, try to be brief.

Question 3:
Which instruction helps the most? Please explain yourself.

Question 4:
Which tricks did you teach yourself to walk better in an exoskeleton? Please explain yourself.
Question 5:

What information about the exoskeleton or yourself would you like to receive while walking in the exoskeleton in order to improve your walking in the exoskeleton? (You can think about feedback about how much you lean to one side, the beginning/ending of a step etcetera.)

Question 6:
What type of feedback do you think is most helpful, vibrotactile, auditory or visual? Vibrotactile feedback: e.g. vibrations to the chest. Auditory feedback: e.g. beep sounds. Visual feedback: e.g. led lights.
O Vibrotactile
O Auditory
O Visual

Question 7:
Do the noises made by the exoskeleton help you during practice? Can you for example hear when the leg is being moved so you know the position of your leg?
O Yes
O No

Question 8:
Imagine you get feedback through vibrations. How much would it help if a vibration would be applied to you when the exoskeleton starts making a step?
O Nothing at all
O Not much
O Neutral
O A little
O A lot
Please explain yourself.

Feedback about gait phases
In Figure 1 a gait cycle is shown, which contains different gait phases. Feedback can be given about the current gait phase (with e.g. changes in intensity of the feedback) while walking in an exokeleton.

Figure 1:

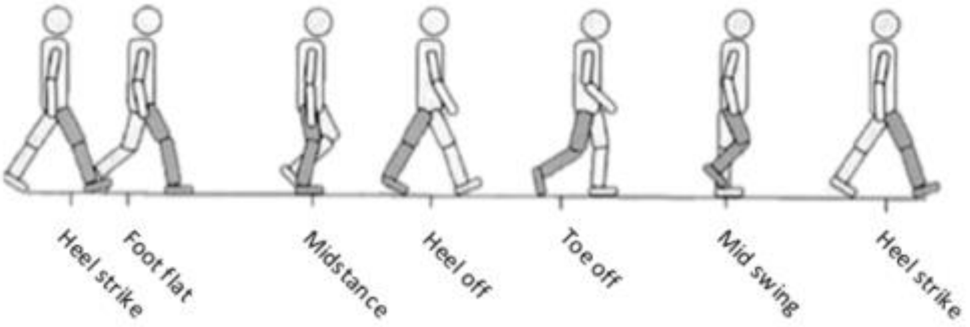

Question 9:
Imagine you get feedback about your gait phases through vibrations. To various gait phases belong various intensities of vibrations. How much would it help if the exoskeleton would give continuous feedback about gait phases? Continuous feedback consists of a continuous signal indicating the current gait phase, see Figure 2. So, you constantly feel a vibration that varies in intensity (increase/decrease) with the present gait phase.

O Nothing at all
O Not much
O Neutral
O A little
O A lot

Please explain yourself.

Figure 2:

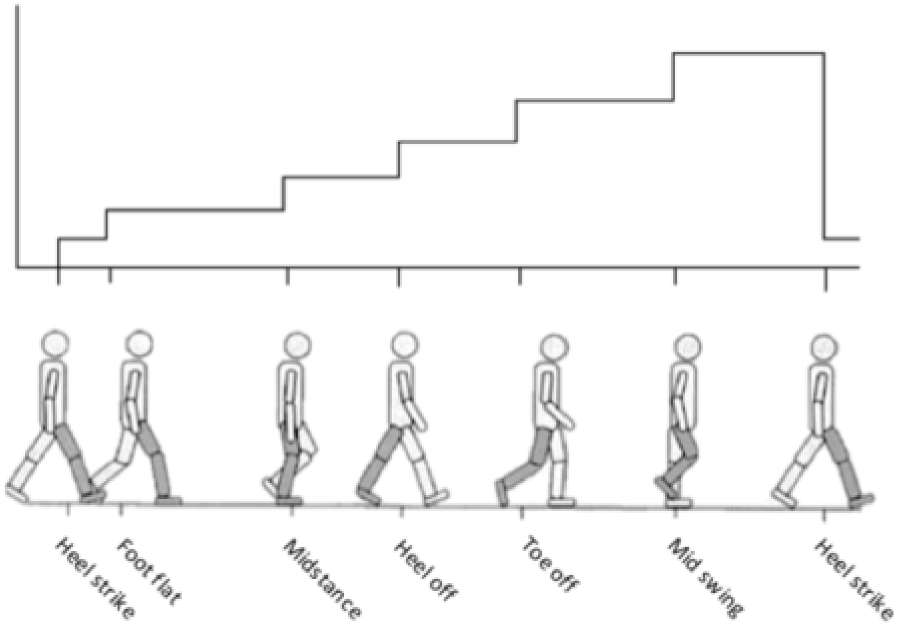

Question 10:
Imagine you get feedback about your gait phases through vibrations. To various gait phases belong various intensities of vibrations. How much would it help if the exoskeleton would give discrete feedback about gait phases? Discrete feedback consists of loose short signals that indicate the beginning of the new gait phase, see Figure 3.
O Nothing at all
O Not much
O Neutral
O A little
O A lot
Please explain yourself.

Figure 3:

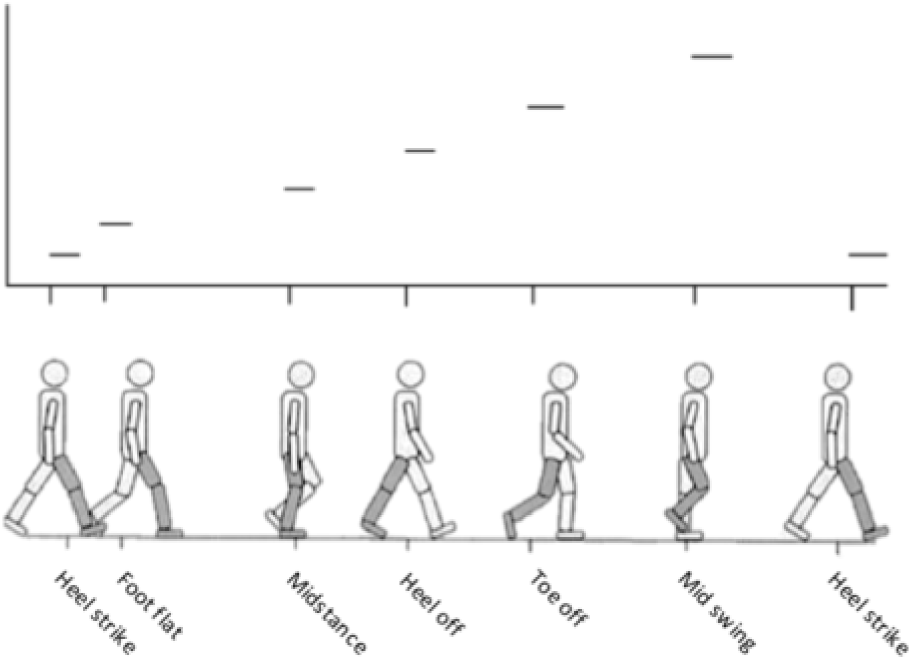

Question 11:
What kind of feedback do you think would be most helpful, continuous or discrete feedback (see Figure 2 and Figure 3)?
O Continuous
O Discrete
Please explain yourself.

Question 12:
About which gait phases do you think it is important to receive feedback (see Figure 4)? You can check multiple boxes.
O Heel strike
O Foot flat

O Midstance
O Heel off
O Toe off
O Mid swing
Please explain yourself.

Figure 4:

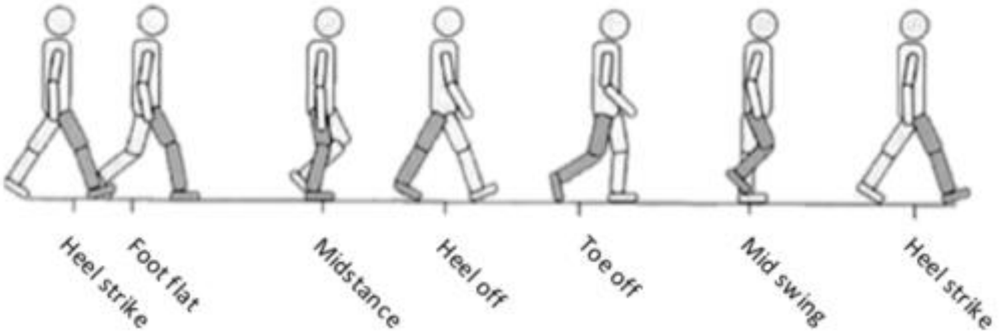

Other information provided by feedback
Question 13:
Imagine you get feedback about your gait phases through vibrations. These vibrations provide information about the position of your foot during a step. The further your foot swings, the higher the intensity of the vibration. How much would it help if the exoskeleton would give feedback on the swing of your foot, from releasing the toes to the touchdown of the heel (see Figure 5)?
O Nothing at all
O Not much
O Neutral
O A little
O A lot
Please explain yourself.

Figure 5:

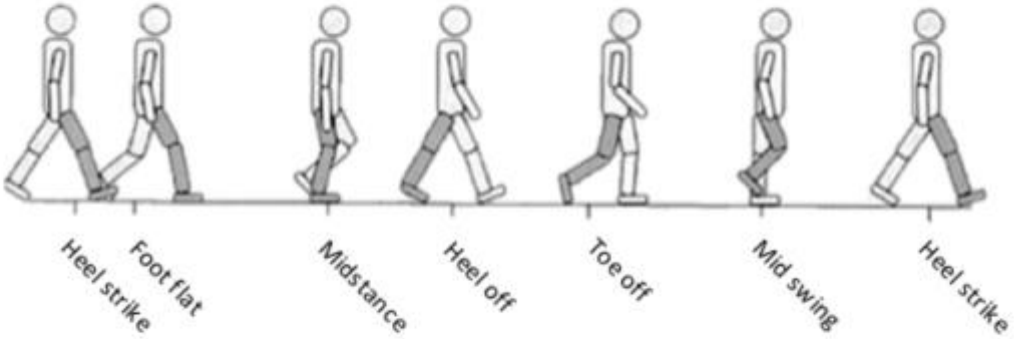

Question 14:
Imagine you get feedback about your gait phases through vibrations. These vibrations provide information about your weight shift from one side to the other side (e.g. from left to right, see Figure 6). When your weight has been moved to one side, the leg can swing forward on the other side. The larger the shift, the higher the intensity of the vibration. How much would this help you in dealing with the exoskeleton?
O Nothing at all
O Not much
O Neutral
O A little
O A lot
Please explain yourself.

Figure 6:

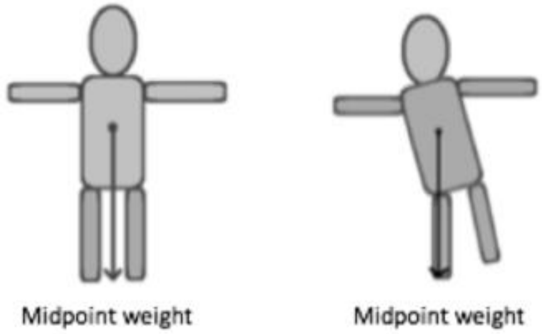

Question 15:
Imagine you get feedback about your gait phases through vibrations. These vibrations provide information about your weight shift from front to back (see Figure 7). The larger the displacement, the higher the intensity of the vibration. How much would this help you in dealing with the exoskeleton?
O Nothing at all
O Not much
O Neutral
O A little
O A lot
Please explain yourself.

Figure 7:

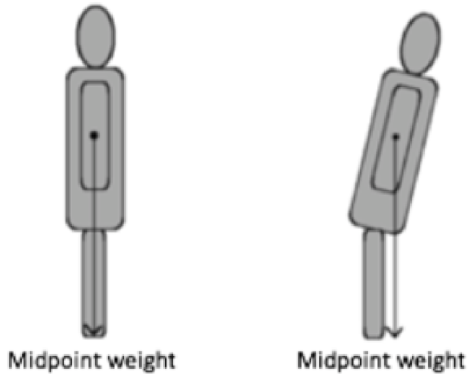

Question 16:
Is there anything you would like to add to this questionnaire? Or have ideas occurred to you while completing this questionnaire, for example on information provided by feedback?
